# Assessing Association Between Circulating Bilirubin Levels and the Risk of Frailty: An Observational and Mendelian Randomization Study

**DOI:** 10.1002/jcsm.13642

**Published:** 2024-11-25

**Authors:** Jun Wu, Jia‐hao Xu, Hao‐qi Zou, Yi‐jiang Ouyang, Shang‐jie Li, Liang Wu, Jie Zhang, Ming‐Juan Yin, Dong‐qing Ye, Jin‐dong Ni

**Affiliations:** ^1^ Department of Epidemiology and Biostastics, School of Public Health, Shunde Women and Children's Hospital Guangdong Medical University Dongguan China; ^2^ School of Public Health Anhui University of Science and Technology Hefei Anhui China; ^3^ Precision Key Laboratory of Public Health Guangdong Medical University Dongguan China

**Keywords:** bilirubin, frailty, Mendelian randomization

## Abstract

**Background:**

Bilirubin is a by‐product of haemoglobin breakdown and has been reported to be a potent antioxidant recently. While elevated levels of bilirubin have been linked to a reduced risk of various diseases, their role remains unknown in frailty. This study aims to explore the relationship between serum bilirubin levels and the risk of frailty.

**Methods:**

This cohort study included 442 223 White British participants (aged 39 to 73 years) with an available frailty index at baseline (2006 to 2010) from the UK Biobank. The associations of total/direct bilirubin levels with the continuous frailty index were analysed by multivariable linear regression, and multivariable logistic regression was used after classifying frailty outcomes into non‐frailty, pre‐frailty and frailty. A Mendelian randomization (MR) analysis was applied to evaluate the association of genetically predicted bilirubin levels with frailty risk.

**Results:**

The prevalence rates of both pre‐frailty and frailty were 46.17% and 12.49%, respectively, with higher rates observed in women than in men (pre‐frailty: 47.33% vs. 44.79%, frailty: 13.64% vs. 11.13%, respectively). There was a non‐linear negative association between total bilirubin levels and frailty indexes (*p* < 0.0001). Mildly elevated total bilirubin levels had protective effects against pre‐frailty (OR = 0.863, 95% CI: 0.849 to 0.879, *p* < 0.001) and frailty (OR = 0.660, 95% CI: 0.641 to 0.679, *p* < 0.001). Increased total bilirubin levels were more beneficial for women with frailty risk (percent changes per SD μmol/L = −0.37%, 95% CI: −0.40% to −0.34%). The MR analysis revealed a negative association between genetically predicted total/direct bilirubin levels and frailty risk (both *p* < 0.0001).

**Conclusions:**

Circulating total/direct bilirubin levels were negatively associated with frailty risk in White British individuals. Mildly elevated total bilirubin levels were more beneficial for women subpopulation.

## 1 Introduction

Frailty is widely considered as one of the most severe public health concerns. Frailty is a complex clinical syndrome characterized by weakness and decline in physiologic reserves across multiple organs and systems, resulting in increased vulnerability to stressors [1]. It exacerbates the incidence of falls, disabilities, premature death, admission to long‐term care and hospitalization of seniors, which places an increased burden on communities and healthcare systems worldwide [1].

Chronic inflammation, oxidative stress and their interactions are risk factors for frailty [2, 3]. Both frail and pre‐frail individuals have elevated levels of oxidative stress markers, pro‐inflammatory cytokines and reduced concentrations of endogenous antioxidants [4]. Based on the catabolic effects of pro‐inflammatory cytokines on the muscles of older adults [5], they might be candidates for preventive treatment with anti‐inflammatory and anti‐oxidative agents. A compelling body of experimental and clinical evidence has demonstrated that serum bilirubin, a degradation product of haem, is an endogenous antioxidant. Mildly elevated serum bilirubin levels have been reported to suppress lipid oxidation and retard sarcopenia in frail individuals [6, 7]. Epidemiological studies have also revealed that a low serum bilirubin level may be a strong predictive biomarker of frailty. Its clinical relevance has been studied as it is closely and favourably connected with the disabilities of daily living in older adults [6, 8]. Similar results have been reported for oxidative stress‐related diseases in humans, such as cancers, stroke and kidney function [9, 10, 11]. Although current studies have put forward these possibilities, it remains unclear whether circulating bilirubin can serve as a predictive biomarker or therapeutic target for modulating frailty status. This is mainly because a causal relationship cannot be deduced from observational studies due to the inevitable presence of confounders and bias, and no clinical trial on the role of bilirubin in frailty has been reported yet.

Mendelian randomization (MR) is an effective method to correct the confounding bias and identify a relationship between environmental and medical factors in complex diseases [12, 13]. MR uses genetic variants associated with an exposure to estimate the effect of genetic predisposition on the disease [12]. Since the randomization for the genetic variants of bilirubin occurs before birth, it is independent of the confounders of the exposure and outcome, and the outcome after conditioning for the exposure [10]. In recent studies, MR has been widely used to explore influencing factors associated with the comorbidities of frailty [14, 15].

In this study, we conducted an observational study to analyse the associations between circulating bilirubin levels and frailty risk in the UK Biobank cohort. Furthermore, the associations of genetically predicted bilirubin levels with frailty risk were tested by the MR and hypothesized that mildly elevated bilirubin levels could be beneficial for frail individuals.

## 2 Methods

### 2.1 Study Population

The UK Biobank, a prospective cohort study, recruited  502 505 participants aged 39 to 73 years from 22 assessment centres across England, Wales and Scotland from 2006 to 2010. The study design has been described previously in detail [16, 17]. Detailed data on the phenotypes and genotypes of each participant were collected through touchscreen questionnaires, health records, physical measurements, biological samples and genome‐wide genotyping. Our study included a total of 442 223 participants. Participants lacking data on the frailty phenotypes were excluded (*n* = 1729); analyses in this study were restricted to individuals whose ethnic background was the White British; therefore, those who were non‐White British were also excluded (*n* = 58 553). Ethics approval for the UK Biobank cohort was obtained from the North West Centre for Research Ethics Committee (11/NW/0382), and signed electronic consent from each participant was obtained at baseline assessments [16].

### 2.2 Ascertainment of Pre‐Frail and Frail Participants

The frailty index was used to define the frailty phenotypes in this study, which is an adapted measure of frailty that has been previously developed and used for participants from the UK Biobank cohort as described elsewhere [18]. More information is available in the Table S1. The frailty index ranges from 0 to 1 and indicates the proportion of the number of deficits to all possible deficits. Using the frailty index criteria, participants were classified into frail (score range: 0.21 to 1.00), pre‐frail (score range: 0.10 to 0.21) and non‐frail (score range: 0 to 0.10) groups [19].

### 2.3 Laboratory Measurement of Circulating Bilirubin

Blood samples were collected at recruitment from each participant and were stored in a fully automated −80°C working archive. Total bilirubin levels were measured using colorimetric assays, and direct bilirubin levels were determined by DPD analysis (Beckman Coulter United Kingdom Ltd. Beckman Coulter AU5800 analyser). The average within‐laboratory coefficients of variation of the assays for low, medium and high internal quality control level samples for total bilirubin and direct bilirubin ranged from 1.48% to 1.92% and 1.73% to 2.60%, respectively.

### 2.4 Covariate Measurements

Adjusted covariates for this study included the following: age; sex; number of individuals in the household; household income categories (< £18 000, £18 000–29 999, £30 000–51 999, £52 000–100 000, and > £100 000); education attainment (degree or no degree); Townsend deprivation index (TDI) [20]; body mass index (BMI, < 25.0 kg/m^2^ or ≥ 25.0 kg/m^2^), which was calculated by weight [kg]/(height [m])^2^ measured at baseline [21]; smoking status (no current smoking or current smoking); alcohol consumption (daily, 1–4 times per week, 1–3 times per month, occasional, or never); regular exercise was defined as 75 mins of vigorous activity or 150 mins of moderate activity or an equivalent combination per week [22]; processed meat intake (times/week); red meat intake (times/week); and fruits and vegetables intake (grams/day). These variables were obtained through questionnaires or laboratory tests.

## 3 Statistical Analysis

### 3.1 Observational Analyses of Associations Between Serum Bilirubin Levels and Frailty Risk

Total and direct bilirubin concentrations exhibit a logarithmic distribution; therefore, all bilirubin levels were represented and analysed as log‐transformed values in this study. Multivariable linear regression models were employed to estimate the associations between serum bilirubin levels and the frailty index, with adjustments for covariates. Subsequently, we built restricted cubic spline (RCS) models to capture the non‐linear dose–response relationships between serum bilirubin levels and frailty risk.

The study population was divided into non‐fail, pre‐fail and frail participants according to frailty index criteria, and multivariable logistic regression models were utilized to estimate the odds ratios (ORs) and 95% confidence intervals (95% CIs) for the associations between serum bilirubin levels and frailty risk. Overall, three different adjusted models were constructed: Model 0 was the crude model; Model 1 was adjusted for age and sex; the fully adjusted model (Model 2) was additionally adjusted for TDI, number of individuals in the household, education attainment, BMI, smoking, alcohol consumption, and regular exercise. The same pattern analyses were repeated for the sex‐based and age‐based subpopulations. The risk of frailty was investigated in the following sensitivity analyses: (i) multivariable logistic regression models additionally adjusted for other covariates, including diets (fruits, vegetables, red meats, and processed meats), household incomes and sleeping time; (ii) the participants without serum bilirubin data were directly excluded rather than imputing missing values by the multiple imputation method; (iii) the participants were reclassified as non‐frailty and frailty; (iv) the definition of frailty was based on five phenotypes (weight loss, exhaustion, grip strength, low physical activity, and slow walking pace).

### 3.2 Linear and Non‐Linear MR Analyses

We performed linear and non‐linear MR analyses to examine the genetic evidence for the associations of serum bilirubin (including total and direct bilirubin) levels with the frailty index. The weighted genetic scores (WGSs) of total/direct bilirubin were used as instrumental variables in our MR, which were validated in the previous study [23]. Briefly, the batch screening iterative lasso (BASIL) algorithm was applied to construct the WGSs, which considers all the genetic variants available in the input dataset and performs variable selection (Tables S2 and S3). The details regarding the WGSs construction were described in the Supplementary Methods. The information on the effects of the genetic variants used as weights during the construction of the WGSs of total/direct bilirubin were obtained from the database (http://www.pgscatalog.org/score/PGS000681 and http://www.pgscatalog.org/score/PGS000697) [24].

For the linear MR analysis, we computed the MR estimates using the ratio of coefficients. Linear regression was used to estimate the association of bilirubin‐WGS with serum bilirubin, as well as the association of bilirubin‐WGS with the frailty index. We adjusted these analyses for sex, age, the single nucleotide polymorphism (SNP) arrays and the top 10 genetic principal components. We also performed stratified analyses in which MR estimates were computed for categories of residual bilirubin concentration (according to quartiles). Residual bilirubin concentration was defined as the participants' serum bilirubin concentration minus the centre genetic contribution by bilirubin‐WGS, which can avoid collider bias.

For non‐linear MR analysis, we used the fractional polynomial method to capture non‐linear genetic associations. Briefly, we stratified our sample into 50 strata using the residuals of serum bilirubin after regressing on bilirubin‐WGS. Within each stratum, we computed the localized average causal effect (LACE), which is the ratio of the coefficient of the bilirubin‐WGS‐frailty index association to that of the bilirubin‐WGS‐bilirubin association. LACE is equivalent to the traditional linear MR estimate within each stratum. We performed a meta‐regression of LACE against stratum‐specific mean bilirubin by fitting a range of fractional polynomial exposure‐outcome models of Degrees 1 and 2 and selecting the best‐fitting model based on the likelihood ratio test. The fractional polynomial test was performed for non‐linearity and compared the best‐fitting fractional polynomial model of Degree 1 against the linear model. Non‐linear MR analyses assume that the association of the genetic variant with exposure is constant over the entire distribution of exposure. To test this assumption, we computed the bilirubin estimate in each of the 50 strata and then examined the heterogeneity among the strata using the trend test and Cochran's *Q* test.

All data collation and analyses in this study were carried out using R software 4.2.2 version. Two‐sided *p* values < 0.05 were considered to be statistically significant.

## 4 Results

### 4.1 Population Characteristics

All data on 442 223 participants were obtained from the UK Biobank cohort for this study; Table 1 shows the basic characteristics of the study population. The mean (SD) age of participants was 56.86 (8.00) years, with 240 096 (54.29%) being women. The cohort included 55 250 (12.49%) frail and 204 155 (46.17%) pre‐frail participants. The prevalence rates of both pre‐frailty and frailty were higher in women compared to men (pre‐frailty: 47.33.% vs. 44.79%; frailty: 13.64% vs. 11.13% respectively). The SD of the baseline log_e_ total bilirubin level was 0.39 μmol/L (equivalent to 1.5‐fold higher total bilirubin level, as e^0.39^ = 1.48). The SD of the baseline log_e_ direct bilirubin level was 0.36 μmol/L (equivalent to a 1.5‐fold increase in the direct bilirubin level, as e^0.36^ = 1.43). Total bilirubin levels were lower in pre‐frail and frail older participants than in non‐frail older participants, but no obvious difference was observed in the direct bilirubin level.

**TABLE 1:** The characteristics of the study population.

	Total participants (442 223)	Study population			Women (240 096)			Men (202 127)		
		Non‐frail (182 818)	Pre‐frail (204 155)	Frail (55 250)	Non‐frail (93 710)	Pre‐frail (113 632)	Frail (32 754)	Non‐frail (89 108)	Pre‐frail (90 523)	Frail (22 496)
Frailty index, median (IQR)	0.112 (0.071, 0.168)	0.066 (0.046, 0.082)	0.143 (0.117, 0.168)	0.255 (0.230, 0.291)	0.066 (0.046, 0.082)	0.143 (0.117, 0.168)	0.255 (0.230, 0.291)	0.066 (0.046, 0.082)	0.143 (0.117, 0.168)	0.255 (0.230, 0.291)
Age, mean (SD), years	56.86 (8.00)	55.94 (8.08)	57.33 (7.94)	58.15 (7.63)	55.64 (8.02)	57.08 (7.84)	58.09 (7.54)	56.25 (8.13)	57.64 (8.05)	58.22 (7.76)
Townsend deprivation index, mean (SD)	−1.52 (2.96)	−1.91 (2.71)	−1.48 (2.95)	−0.37 (3.41)	−1.93 (2.66)	−1.54 (2.89)	−0.53 (3.32)	−1.89 (2.75)	−1.42 (3.02)	−0.14 (3.51)
Number of individuals in household	2.38 (1.26)	2.50 (1.23)	2.34 (1.24)	2.15 (1.39)	2.47 (1.22)	2.30 (1.21)	2.13 (1.40)	2.54 (1.24)	2.38 (1.27)	2.18 (1.38)
Education										
Degree	137 658	67 987	59 203	10 468	33 423	31 802	6242	34 564	27 401	4226
No degree	304 565	114 831	144 952	44 782	60 287	81 830	26 512	54 544	63 122	18 270
Body mass index										
< 25.0 kg/m^2^	145 963	72 582	62 360	11 021	46 106	41 931	7443	26 476	20 429	3578
≥ 25.0 kg/m^2^	296 260	110 236	141 795	44 229	47 604	71 701	25 311	62 632	70 094	18 918
Smoking status										
No current smoking	396 918	168 196	182 632	46 090	87 621	103 328	28 027	80 575	79 304	18 063
Current smoking	45 305	14 622	21 523	9160	6089	10 304	4727	8533	11 219	4433
Alcohol frequency										
Daily	93 053	41 218	42 987	8848	17 209	18 931	3920	24 009	24 056	4928
1–4 times per week	222 728	99 668	101 112	21 948	49 628	53 577	11 792	50 040	47 535	10 156
1–3 times per month	49 463	18 588	23 596	7279	11 432	15 430	4764	7156	8166	2515
Occasional or never	76 979	23 344	36 460	17 175	15 441	25 694	12 278	7903	10 766	4897
Regular exercise										
Yes	296 843	131 868	135 418	29 557	62 020	69 140	16 281	69 848	66 278	13 276
No	145 380	50 950	68 737	25 693	31 690	44 492	16 473	19 260	24 245	9220
Total bilirubin, median (IQR, μmol/L)	8.07 (6.43, 10.41)	8.35 (6.68, 10.74)	7.98 (6.37, 10.30)	7.46 (5.95, 9.64)	7.51 (6.11, 7.48)	7.21 (5.87, 9.08)	6.80 (5.54, 8.57)	9.31 (7.53, 11.84)	9.09 (7.30, 11.59)	8.59 (6.84, 11.07)
Direct bilirubin, median (IQR, μmol/L)	1.51 (1.21, 1.99)	1.54 (1.23, 2.02)	1.50 (1.20, 1.98)	1.44 (1.19, 1.91)	1.38 (1.15, 1.76)	1.33 (1.13, 1.70)	1.31 (1.12, 1.64)	1.74 (1.40, 2.24)	1.75 (1.39, 2.26)	1.71 (1.35, 2.23)

### 4.2 Association Between Total Bilirubin and Frailty Risk

**FIGURE 1:**
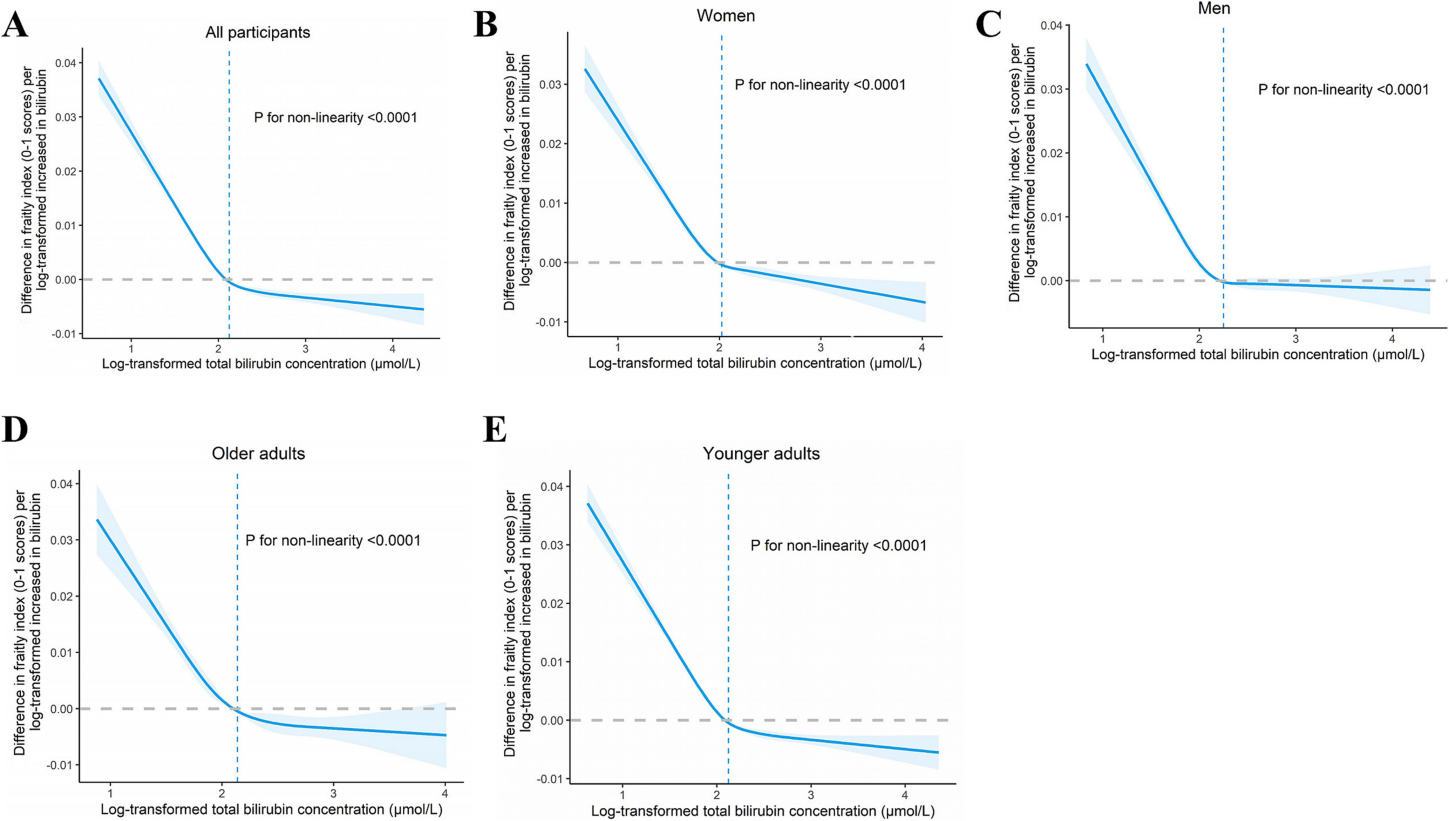
The non‐linear dose–response relationships between circulating total bilirubin levels and frailty indexes.

The risk of frailty increased with low total bilirubin levels. In UK Biobank cohort, there was a non‐linear negative correlation between total bilirubin levels (log‐transformed) and frailty indexes in both the crude and multivariable adjusted models (*p* for non‐linearity < 0.0001) (Figure 1A). The risk of frailty decreased from quintile 1 to quintile 4 of the total bilirubin levels (*p*_trend_ < 0.001), and this association remained robust after adjustment for covariates (Table S4).

**FIGURE 2:**
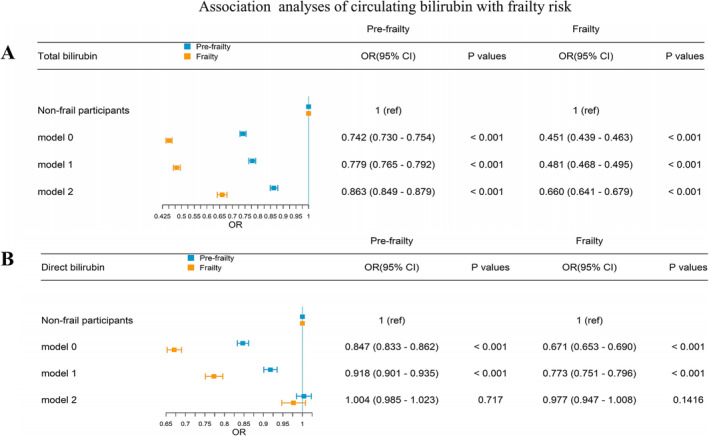
Associations of circulating bilirubin levels with the risk of frailty across frailty categories. Model 0 was the crude model; Model 1: Adjusted for sex and age; Model 2: Model 1 + Townsend deprivation index, number of individuals in the household, education attainment, body mass index, smoking status, alcohol consumption and regular exercise.

When participants were grouped into non‐frail, pre‐frail and frail groups in accordance with the frailty index, total bilirubin levels were associated with frailty risk in participants with pre‐frailty (OR = 0.863, 95% CI: 0.849 to 0.879, *p* < 0.001) and frailty (OR = 0.660, 95% CI: 0.641 to 0.679, *p* < 0.001) (Figure 2A). These results did not change after adjustment for covariates, including sex, age, education, TDI, number of individuals in the household, regular exercise, smoking, alcohol consumption and BMI, indicating that the association between low total bilirubin level and frailty risk was robust. Similar patterns were observed in sensitivity analyses (Table S5): covariates in these analyses included dietary factors (fruits, vegetables, red meats, and processed meats), household incomes and sleeping time; participants with incomplete bilirubin data were excluded in study population; frailty was defined based on five phenotypes (weight loss, exhaustion, grip strength, low physical activity and slow walking pace); the participant categories were redefined as non‐frailty and frailty. Additionally, total bilirubin levels exert a greater effect in frailty syndrome than in a single specific deficit (Table S6).

### 4.3 Association Between Direct Bilirubin and Frailty Risk

A statistically significant association was observed between direct bilirubin levels (log‐transformed) and the frailty index with or without adjustment for sex and age (both *p* < 0.001) (Figure S1A). Associations between direct bilirubin levels and frailty risk across non‐frail, pre‐frail and frail groups are presented in Figure 2B, varying with model adjustments for different covariates. In crude Model 0 and Model 1 adjusted for sex and age, direct bilirubin levels demonstrated protective effects on pre‐frailty and frailty (both *p* < 0.001); however, the effect became non‐significant after adjusting for education, TDI, number of individuals in the household, regular exercise, smoking, alcohol consumption and BMI (both *p* > 0.05). This was probably caused by confounders or an estimation bias. Similar patterns were observed in the sensitivity analyses (Table S5).

### 4.4 Subgroup Analyses of the Effect of Bilirubin on Frailty Risk

The results of the subgroup analyses are detailed in Table 2. Overall, the frailty index was comparatively higher in women and older participants than in men and younger participants (sex: 0.12 vs. 0.11; age: 0.13 vs. 0.11). In contrast, the bilirubin levels (log‐transformed) were relatively lower in women and younger participants than in men and older participants (total bilirubin, sex: 1.98 vs. 2.21, age: 2.08 vs. 2.10; direct bilirubin, sex: 0.30 vs. 0.55, age: 0.41 vs. 0.42). Similarly, total bilirubin appeared to particularly benefit women with frailty (*p* for interaction = 2.27 × 10^−7^), and the frailty index increased by −0.37% (95% CI: −0.40% to −0.34%) for per SD increase in total bilirubin levels. Direct bilirubin provided modestly protective effects for frailty in women and younger participants (both *p* < 0.001); however, the frailty index increased by 0.06% (95% CI: 0.03% to 0.09%) for per SD increase in direct bilirubin levels of men, and the frailty index increased by 0.16% (95% CI: 0.10% to 0.22%) for per SD increase in direct bilirubin levels of older participants. RCS models were used to determine the non‐linear dose–response relationships between total/direct bilirubin levels and the frailty index in each subgroup (Figures 1 and S1).

**TABLE 2:** Whether the circulating bilirubin effects on frailty risk in the whole cohort is homogeneous across subpopulations.

Participants	Total bilirubin			Direct bilirubin		
	Percent changes (%) and 95% CI per 1 SD	*p*	*p* for interaction	Percent changes (%) and 95% CI per 1 SD	*p*	*p* for interaction
Age subgroup			0.115			1.63 × 10^−15^
≤ 65 years	−0.35 (−0.37, −0.33)	< 2.00 × 10^−16^		−0.04 (−0.06, −0.01)	1.20 × 10^−3^	
> 65 years	−0.35 (−0.40, −0.29)	< 2.00 × 10^−16^		0.16 (0.10, 0.22)	3.97 × 10^−8^	
Sex subgroup			2.27 × 10^−7^			7.07 × 10^−14^
Men	−0.29 (−0.32, −0.26)	< 2.00 × 10^−16^		0.06 (0.03, 0.09)	3.47 × 10^−5^	
Women	−0.37 (−0.40, −0.34)	< 2.00 × 10^−16^		−0.07 (−0.10, −0.04)	1.88 × 10^−7^	

*Note:* Adjustment for age, sex, Townsend deprivation index, number of individuals in the household, household incomes, education attainment, body mass index, smoking status, alcohol consumption and regular exercise.

### 4.5 Genetically Predicted Bilirubin Levels and Frailty Risk

**FIGURE 3:**
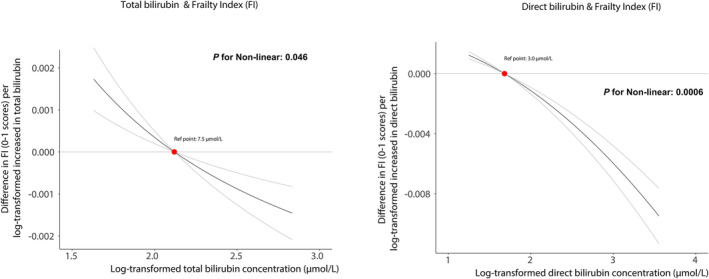
Genetic associations of circulating bilirubin concentrations with frailty indexes.

As shown in Figure 3, genetically predicted total bilirubin levels had an approximately linear association (*p* for nonlinearity = 0.046), when displayed across the measured total bilirubin concentrations (log‐transformed) as shown on the x‐axis. Genetically predicted total bilirubin levels were negative association with the frailty index in participants with measured total bilirubin concentrations. Likewise, in the stratified analyses, we also observed an association between genetically predicted total bilirubin levels and the frailty index from the participants with the lowest (quartile 1) and highest (quartile 4) measured total bilirubin concentrations (−0.000937 and −0.000999 in frailty indexes per log‐transformed increase in the total bilirubin, *p =* 0.0003 and *p =* 2.19 × 10^−5^, respectively) (Table S7 and Figure S2). For the association of genetically predicted direct bilirubin with the frailty index, there was a significant frailty index curvature (*p* for nonlinearity = 0.0006). Genetically predicted direct bilirubin had an inverse association with the frailty index in participants with measured direct bilirubin concentrations (Figure 3). Besides, significant effects of increasing genetically predicted direct bilirubin levels on the frailty index from participants in quartiles 1, 2 and 4 of measured direct bilirubin concentrations were also observed (Table S7 and Figure S2). The results of linear MR estimates on both total bilirubin and direct bilirubin were robust in MR‐Egger and weighted median (Table S8).

## 5 Discussion

In this large‐scale population‐based cohort study, we performed observational and MR analyses to assess the associations between circulating total/direct bilirubin levels and frailty risk for the first time. Our results confirmed earlier reports of a higher frailty index in women and older individuals. Additionally, we found a negative association between bilirubin levels and frailty risk in women subpopulation.

Previous studies have suggested that mildly elevated bilirubin levels are associated with an increased lifespan [25, 26]. This stimulated interest in the relationship of bilirubin levels with age‐associated diseases [6, 27, 28, 29]. Subsequently, the associations of bilirubin with crucial characteristics of frailty were reported including sarcopenia [27], disability [6], comorbidities [28] and cognitive dysfunction [29]. Frailty is a multisystem disorder that increases with age [1]. One study proposed that a low bilirubin level could be considered as a strong predictor of disabilities in daily living activities in frail participants with diabetes [6]. The odds of functional dependence increased with decreasing levels of bilirubin among older individuals [30]. Systematic meta‐analyses also demonstrated that mildly elevated bilirubin levels have protective effects on oxidative stress‐related comorbidities of frailty such as diabetes, diabetic vascular complications, chronic kidney diseases, atherosclerotic diseases and Alzheimer's disease [31]. In addition, bilirubin levels were demonstrated to be positively associated with muscle mass among participants with sarcopenia [27]. Based on the above findings, bilirubin can be considered a potential preventive agent for frailty, although further studies are still needed considering the small sample sizes and unknown confounding factors in previous studies.

Consistent with previous studies, this study also found that a lower total bilirubin level was associated with a higher risk of frailty. To our knowledge, our study is the first to investigate this association for whole‐body frailty, which is different from previous studies that merely focused on a single frail phenotype or symptom of frailty, and we found that bilirubin levels may exert a greater protective effect in whole‐body frailty than in single deficit states. One previous study on biomarker screening for frailty found low bilirubin levels in pre‐frail and frail individuals, indirectly verifying our robust findings [8]. In the subpopulation analysis, we also noted that circulating bilirubin levels were more beneficial for women, which was in line with other diseases, indicating that bilirubin's antioxidant effect has a protective effect against frailty in women [32]. We are not aware of any other large‐scale cohort studies that have previously been reported on the relationship between bilirubin and frailty. This study ensured an adequate sample size to avoid selection bias. Furthermore, we took into account confounding bias, and accordingly adjusted the multiple covariate models (Models 0–2). We used RCS to evaluate the dose–response effect of bilirubin on frailty as a continuous scale in the overall population. Unlike categorization, the method we used to fit the model took advantage of the full information available and flexibly captured the dose–response relationship and even non‐linearity. It has been reported earlier that a higher bilirubin level was significantly related to a lower incidence rate of chronic obstructive pulmonary disease [33], while a bilirubin concentration of 10.0 μM was the cut‐off value for cardiovascular disease risk [34], and these diseases are considered as risk factors for frailty. The above evidence confirms the beneficial effects of mildly elevated total bilirubin concentrations on participants with frailty.

Another point worth highlighting is that this study is the first to use a MR analysis to test the relationships between genetically predicted bilirubin levels and frailty risk. We found that genetic predisposition for a higher bilirubin level is associated with a lower frailty index, which was in line with the findings of our observational study. This approach also can make up for the shortcomings of the observational study, such as the presence of confounding variables [35]. Moreover, a large sample size of 442 223 individuals from the UK Biobank database was used for this large‐scale analysis. Similarly, the possible causal relationships between mildly elevated bilirubin levels and other age‐associated diseases were also illustrated in several MR studies on stroke [10] and chronic kidney disease [11]; individuals with these diseases are prone to developing frailty, especially older individuals [36]. Thus, there is substantial evidence that mildly elevated bilirubin levels may play a protective role in frail individuals.

Recent research on bilirubin has profoundly revealed its biological functions, including immune‐modulatory, anti‐mutagenic, potent antioxidant and lipid‐lowering effects on multiple organs and systems in humans when present at mildly elevated levels [37, 38]. Evidence from meta‐analyses supports that mildly elevated bilirubin, caused by genetic mutations, is clearly associated with a decreased risk of developing cardiovascular diseases (CVD), as seen in Gilbert's syndrome, Rotor syndrome and Dubin‐Johnson syndrome [39, 40]. This may be related to bilirubin's ability to inhibit low‐density lipoprotein oxidation and neutralize free radicals [39, 40]. Large longitudinal and epidemiological studies have indicated that patients with Gilbert's syndrome and elevated bilirubin levels experience reduced rates of cancer, CVD and all‐cause mortality compared to healthy individuals [41, 42]. Similarly, carriers of genetic mutations associated with bilirubin levels in the Framingham Heart Study also showed a lower risk of CVD [43], providing clinically translational models that underscore the physiological importance of mildly elevated bilirubin in disease prevention [39, 43, 44, 45]. Furthermore, emerging studies have investigated interventions to manipulate bilirubin levels in both in vivo and in vitro models. Single‐dose administration of bilirubin was shown to reduce tissue injury in mouse models of endotoxemia, suggesting that bilirubin may help mitigate hyperinflammatory reactions [46]. Additionally, increased plasma bilirubin levels were found to improve fatty liver disease and blood glucose levels in leptin‐deficient obese mice [47]. Bilirubin has been shown to scavenge free radicals, normalize blood pressure in angiotensin II‐dependent hypertensive mice [38, 48] and prevent endothelial dysfunction by inhibiting the expression of inflammatory cytokines through the suppression of transcription factors such as NF‐kappaB [38, 49]. Furthermore, novel studies have reported the use of bilirubin nanoparticles to treat microbiome‐associated disorders, obesity and nonalcoholic fatty liver disease [50, 51, 52, 53]. Frailty is a clinical syndrome characterized by a decline in multiple physiological systems, commonly associated with immune inflammation, endocrine disorders, oxidative stress, and so forth. Therefore, bilirubin may be an important influential factor affecting the occurrence and development of frailty and modulating bilirubin levels appears to be a promising potential strategy for the prevention of frailty.

### 5.1 Strengths and Limitations of This Study

Strengths of the study include a large sample size, a standardized protocol for data collection and the ascertainment of outcomes by using the comprehensive frailty index, collectively leading to analyses with sufficient statistical power. Furthermore, controlling the confounding factors and sensitivity analyses increased the likelihood of obtaining accurate and reproducible results in the observational study. To the best of our knowledge, this study is the first to perform a MR analysis to evaluate the genetically predicted bilirubin levels on frailty risk. However, this study has several limitations: First, the analyses were performed only on individuals of the White British descent; thus, these findings should be demonstrated among other ethnic groups in future studies for improving the diversity and representativeness of the sample. Second, repeat measurements of bilirubin levels were not performed to correct for the regression dilution bias. Third, the extent to which mildly elevated bilirubin levels are beneficial for participants with frailty cannot be answered in this study, moreover its applications in clinical practice and its biological mechanisms for frailty also need further explorations and validations. Fourth, this study utilized non‐linear MR to examine the link between genetically predicted bilirubin levels and frailty index. This technique, an extension of traditional MR, conducts distinct instrumental variable analyses across population strata with differing mean exposure levels, based on the conventional residual method for stratifying the population assumes a linear and uniform effect of the genetic instrument on exposure. Recent methodological advances have provided other non‐linear MR approaches like the doubly‐ranked stratification method [54]. It is also worth noting that as GWAS findings on bilirubin levels and frailty continue to evolve. Future validation with the doubly‐ranked stratification method and updated GWAS summary statistics will be crucial to substantiate our results and refine our understanding of this relationship.

In conclusion, a lower bilirubin level is associated with a higher frailty risk, and mildly elevated bilirubin levels might be beneficial for frailty improvement in the White British population. Furthermore, circulating bilirubin has a more protective effect against frailty in women. A genetic predisposition for mildly elevated serum bilirubin levels may be a protective factor for frailty. These findings highlight an important potential biological marker involved in frailty, and additional studies are warranted to generalize the findings to the worldwide population and explore the mechanisms linking bilirubin to frailty.

## Ethics Statement

The North West Multi‐Centre Research Ethics Committee approved the collection and use of UK Biobank data (11/NW/0382), and all participants signed the electronic consent.

## Conflicts of Interest

The authors declare no conflicts of interest.

## Supporting information


**Figure S1** The non‐linear dose–response relationships between direct bilirubin and frailty indexes.
**Figure S2.** The check of the MR analysis.
**Table S1**. Feature items used to build the frailty index.
**Table S4**. Association of total bilirubin levels with frailty risk.
**Table S5**. Sensitivity analyses of the associations between bilirubin and frailty risk.
**Table S6**. Association analyses of circulating bilirubin and the specific deficit risk.
**Table S7**. Linear Mendelian randomization analyses of genetically predicted bilirubin and overall and by stratified residual measured bilirubin categories.
**Table S8**. Linear Mendelian randomization estimates from robust methods (MR‐Egger and weighted median).

**Table S2** Genetic variants used to construct weighted genetic scores for total bilirubin.

**Table S3** Genetic variants used to construct weighted genetic scores for direct bilirubin.

## Data Availability

Data from UK Biobank are available on application at www.ukbiobank.ac.uk.
