# Supplementary material for: Assessing Association Between Circulating Bilirubin Levels and the Risk of Frailty: An Observational and Mendelian Randomization Study
Source: J Cachexia Sarcopenia Muscle. 2024 Nov 25;16(1):1–11. doi: 10.1002/jcsm.13642 (PMC11670173; doi:10.1002/jcsm.13642)
Supplement: Supplementary file 1 — Figure S1 The non‐linear dose–response relationships between direct bilirubin and frailty indexes. Figure S2. The check of the MR analysis. Table S1. Feature items used to build the frailty index. Table S4. Association of total bilirubin levels with frailty risk. Table S5. Sensitivity analyses of the associations between bilirubin and frailty risk. Table S6. Association analyses of circulating bilirubin and the specific deficit risk. Table S7. Linear Mendelian randomization analyses of genetically predicted bilirubin and overall and by stratified residual measured bilirubin categories. Table S8. Linear Mendelian randomization estimates from robust methods (MR‐Egger and weighted median). [file JCSM-16--s003.doc]

**Assessing causal association between circulating bilirubin levels and the risk of frailty: An observational and** **Mendelian randomization study**

**Supplementary Materials**

**The supplementary materials include:**

1. **Supplementary methods**
2. **Supplementary igures 1-2**

Supplementary figure 1. The non-linear dose–response relationships between direct bilirubin and frailty index.

Supplementary figure 2. The check of the MR analysis.

1. **Supplementary tables 1-4**

Supplementary table 1. Feature items used to build the frailty index.

**Supplementary table 2. Genetic variants used to construct weighted genetic scores for total bilirubin.**

**Supplementary table 3. Genetic variants used to construct weighted genetic scores for direct bilirubin.**

Supplementary table 4 . Association of total bilirubin levels with frailty risk.

Supplementary table 5. Sensitivity analyses of associations between bilirubin and frailty risk.

Supplementary table 6. Association analyses of circulating bilirubin and specific deficit risk

Supplementary table 7. Linear Mendelian randomization analyses of genetically predicted bilirubin and overall and by stratified residual measured bilirubin categories.

**Supplementary table 8. Linear Mendelian randomization estimates from robust methods (MR‐Egger and weighted median)**

**Supplementary references**

1. **Supplementary methods**

**Detail regarding the polygenic risk scores of the bilirubin calculation in this study**

We used the weighted genetic scores (WGSs) as instrumental variables (IVs) in all our Mendelian randomization analyses. All genetic variants used to construct WGSs for bilirubin in this study were listed in **Supplementary table 1-2**, which were developed in UK biobank and validated in both UK Biobank and other external cohorts as the previous study described^[1]^. Briefly, to construct the WGSs for each of the traits, the authors applied the BASIL algorithm implemented in the R package “snpnet”, which is capable of finding the exact solution for L1-penalized multivariate regression (lasso) on an ultrahigh-dimensional large dataset, and considers all of the genetic variants available in the input dataset and performs variable selection and multivariate regression fit simultaneously. Then they randomly split white British individuals into training (70%), validation (10%), and test (20%) sets, using the training and validation sets to optimize the model's sparsity parameter for predictive performance (R²). The (R²=0.33072, Spearman ρ =0.453) & (R²=0.44377 , Spearman's ρ =0.533) of WGSs are for direct and total bilirubin, respectively.

All genetic variants used in the WGSs, are coded as 0, 1 or 2, corresponding to the number of copies of the effect allele (defined as the bilirubin-increasing allele). The weighted allele score for individual i is defined as:


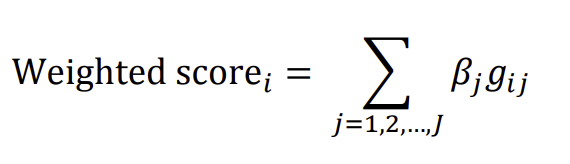


where j indexes the genetic variants (from 1 to J), β j are the weights, taken as the beta coefficient associations from GWAS^[1]^, and gi j = 0, 1, 2 is the number of bilirubin increasing alleles.

**Reference:**

[1] Sinnott-Armstrong N, Tanigawa Y, Amar D, Mars N, Benner C, Aguirre M, et al. Genetics of 35 blood and urine biomarkers in the UK Biobank. Nat Genet. 2021 Feb;53(2):185-194.


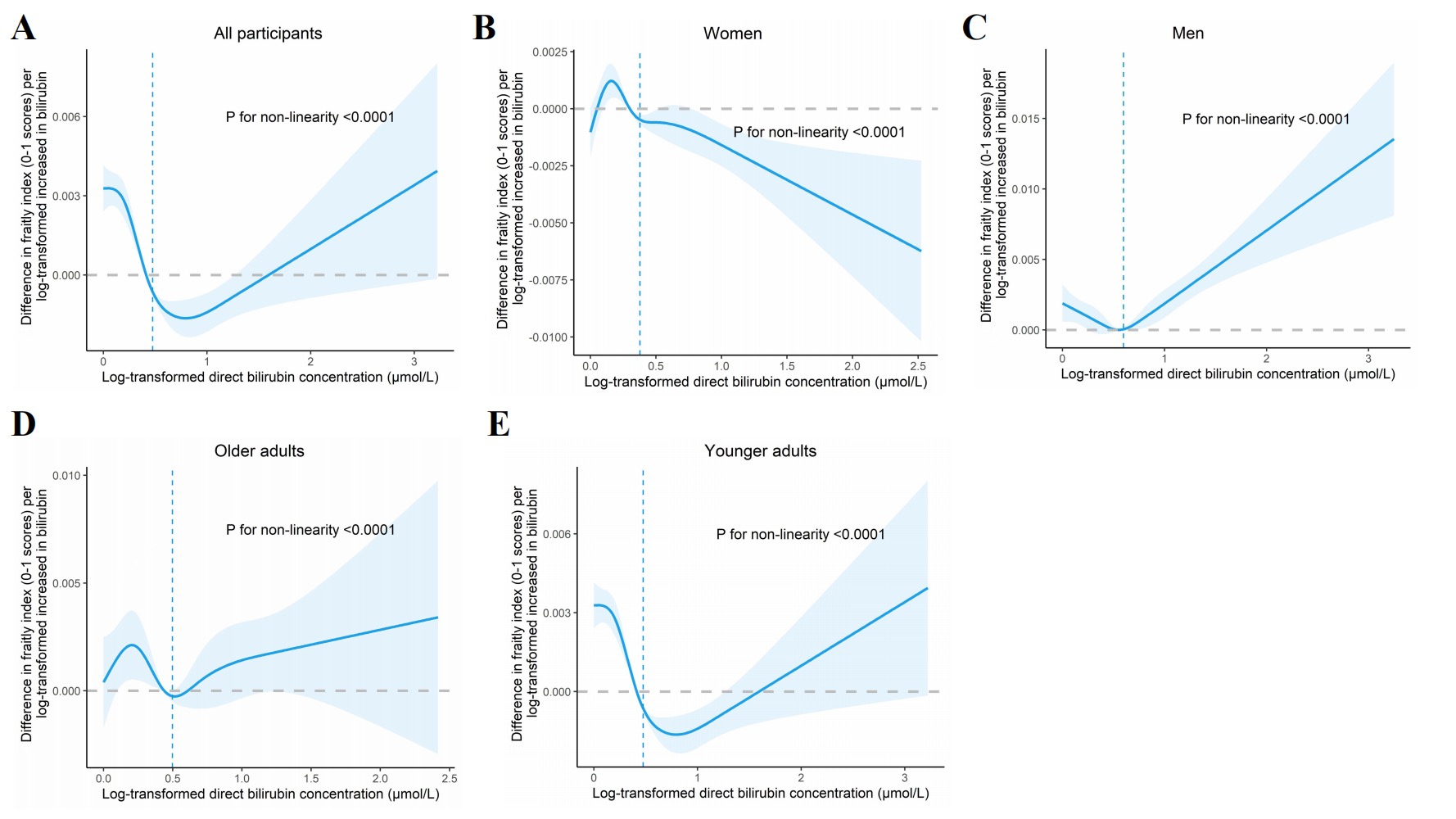
**Supplementary figures**

**Supplementary figure 1 The non-linear dose-response relationships between direct bilirubin and frailty index.**

**
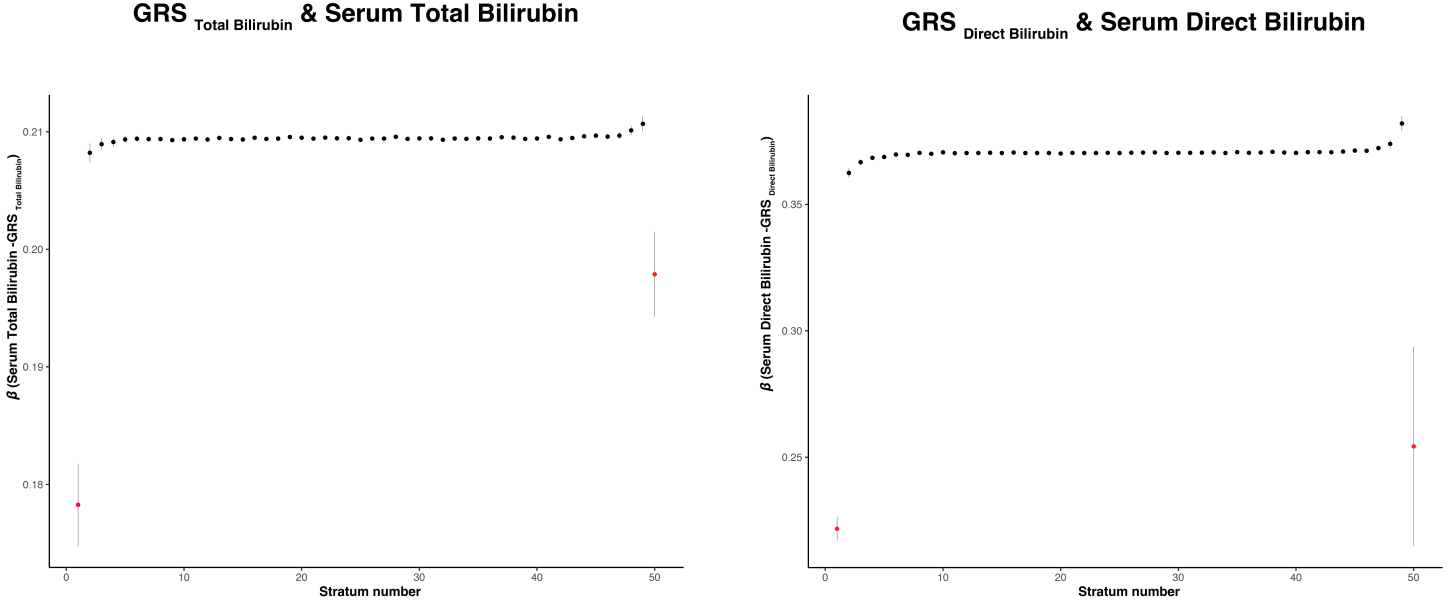
**

**Supplementary figure 2 The check of the Mendelian randomization analyses**

**Supplementary tables**

**Supplementary table1. Feature items used to build the frailty index**.

| **Codes** | **Items** | | **UDL** | **Categories** |
| --- | --- | --- | --- | --- |
| 1 | Sensory | Glaucoma * | 20002-0.0/135-0.0 | no, yes |
| 2 |  | Cataracts * | 20002-0.0/135-0.0 | no, yes |
| 3 |  | Hearing difficulty | 2247-0.0 | no, yes, completely deaf |
|  |  |  |  |  |
| 4 | Cranial | Migraine * | 20002-0.0/135-0.0 | no,yes |
| 5 |  | Dental problems | 6149-0.0 | ulcers, painful gums, bleeding gums, loose teeth, toothache, dentures |
| 6 | Mental wellbeing | Self-rated health | 2178-0.0 | excellent, good, fair, poor |
|  |  |  |  |  |
| 7 |  | Fatigue: frequency of tiredness / lethargy in last two weeks | 2080-0.0 | not at all, several days, more than half, nearly every day |
| 8 |  | Sleep: experience of sleeplessness/insomnia | 1200-0.0 | never/rarely, sometimes, usually |
| 9 |  | Depressed feelings: frequency in last two weeks | 2050-0.0 | not at all, several days, more than half, nearly every day |
| 10 |  | Self-described nervous personality | 1970-0.0 | no, yes |
| 11 |  | Severe anxiety/ panic attacks * |  | no, yes |
| 12 |  | Common to feel loneliness | 2020-0.0 | no, yes |
| 13 |  | Sense of misery (ever/never) | 1930-0.0 | no, yes |
| 14 | Infirmity | Infirmity: long-standing illness or disability | 2188-0.0 | no, yes |
| 15 |  | Falls in last year | 2296-0.0 | categorical: no falls, one fall, more than one |
| 16 |  | Fractures/broken bones in last five years | 2463-0.0 | no, yes |
| 17 | Cardiometabolic | Diabetes * | 20002-0.0/135-0.0 | no, yes |
| 18 |  | Myocardial infarction * | 20002-0.0/135-0.0 | no, yes |
| 19 |  | Angina * | 20002-0.0/135-0.0 | no, yes |
| 20 |  | Stroke * | 20002-0.0/135-0.0 | no, yes |
| 21 |  | High blood pressure * | 20002-0.0/135-0.0 | no, yes |
| 22 |  | Hypothyroidism * | 20002-0.0/135-0.0 | no, yes |
| 23 |  | Deep-vein thrombosis * | 20002-0.0/135-0.0 | no, yes |
| 24 |  | High cholesterol * | 20002-0.0/135-0.0 | no, yes |
| 25 | Respiratory | Breathing: wheeze in last year | 2316-0.0 | no, yes |
| 26 |  | Pneumonia * | 20002-0.0/135-0.0 | no, yes |
| 27 |  | Chronic bronchitis/emphysema * | 20002-0.0/135-0.0 | no, yes |
| 28 |  | Asthma * | 20002-0.0/135-0.0 | no, yes |
| 29 | Musculoskeletal | Rheumatoid arthritis * | 20002-0.0/135-0.0 | no, yes |
| 30 |  | Osteoarthritis * | 20002-0.0/135-0.0 | no, yes |
| 31 |  | Gout * | 20002-0.0/135-0.0 | no, yes |
| 32 |  | Osteoporosis * | 20002-0.0/135-0.0 | no, yes |
| 33 | Immunological | Hay fever, allergic rhinitis or eczema * | 6152-0.0 | no, yes |
| 34 |  | Psoriasis * | 20002-0.0/135-0.0 | no, yes |
| 35 | Cancer | Any cancer diagnosis * | 134-0.0 | no, yes |
| 36 |  | Multiple cancers diagnosed (number reported) | 134-0.0 | Range from 0 to 6 |
| 37 | Pain | Chest pain | 2335-0.0 | no, yes |
| 38 |  | Head and/or neck pain | 6159-0.0 | no, yes (combining responses to pain in head and neck/shoulders) |
| 39 |  | Back pain | 6159-0.0 | no, yes |
| 40 |  | Stomach/abdominal pain | 6159-0.0 | no, yes |
| 41 |  | Hip pain | 6159-0.0 | no, yes |
| 42 |  | Knee pain | 6159-0.0 | no, yes |
| 43 |  | Whole-body pain | 6159-0.0 | no, yes |
| 44 |  | Facial pain | 6159-0.0 | no, yes |
| 45 |  | Sciatica * | 20002-0.0/135-0.0 | no, yes |
| 46 | Gastrointestinal | Gastric reflux * | 20002-0.0/135-0.0 | no, yes |
| 47 |  | Hiatus hernia * | 20002-0.0/135-0.0 | no, yes |
| 48 |  | Gall stones * | 20002-0.0/135-0.0 | no, yes |
| 49 |  | Diverticulitis * | 20002-0.0/135-0.0 | no, yes |

**Supplementary table 2 and table3 are shown in the excel files.**

**Supplementary table 4. Association of total bilirubin levels with frailty risk.**

| **Frailty Index** | **OR (95% CI)** | ***P* values** | ***P* values for trend** |
| --- | --- | --- | --- |
| **Quintile 1 (lowest)** | reference |  | <0.001 |
| **Quintile 2** | -0.052( -0.076 , -0.027 ) | 4.62×10^-5^ |  |
| **Quintile 3** | -0.076( -0.101 , -0.050 ) | 8.12×10^-9^ |  |
| **Quintile 4** | -0.084( -0.110 , -0.057 ) | 6.06×10^-10^ |  |

Notes: Adjustment for age,sex, Townsend deprivation index (TDI), number of individuals in the household, education ,body mass index (BMI), smoking, alcohol consumption, and regular exercise.

**Supplementary table 5. Sensitivity analyses of associations between bilirubin levels and frailty risk.**

|  | **Covariates additionally adjusted covariates included diet (fruits, vegetables, red meat, process meat), economic (household incomes), and sleep time** | | **The study population was reclassified as non-frailty and frailty** | | **The study population directly excluded rather than** **imputing missing value by multiple impute method about bilirubin levels** | | **Definition of frailty was based on five criteria (weight loss, exhaustion, grip strength, low physical activity, slow walking pace)** | |
| --- | --- | --- | --- | --- | --- | --- | --- | --- |
|  | **Percent changes (%) and 95% CI Per 1 SD** | ***P*** | **OR (95% CI)** | ***P*** | **Percent changes (%) and**  **95% CI Per 1 SD** | **P** | **OR (95% CI)** | ***P*** |
| **Total bilirubin** |  |  |  |  |  |  |  |  |
| **All population** | -0.39%(-0.40%,-0.37%) | < 2.00×10^-16^ | 0.826 (0.812,0.840) | < 2.00×10^-16^ | -0.66%(-0.72%,-0.59) | < 2.00×10^-16^ | 0.831 (0.816,0.846) | < 2.00×10^-16^ |
| **Older adults** | -0.38%(-0.43%,-0.33%) | < 2.00×10^-16^ | 0.849 (0.812,0.888) | < 2.00×10^-16^ | -0.80%(-0.97%,-0.63) | < 2.00×10^-16^ | 0.808 (0.771,0.847) | < 2.00×10^-16^ |
| **Women adults** | -0.34%(-0.37%,-0.31%) | < 2.00×10^-16^ | 0.794 (0.775,0.812) | < 2.00×10^-16^ | -0.24%(-0.28%,-0.21%) | < 2.00×10^-16^ | 0.781 (0.76,0.803) | < 2.00×10^-16^ |
| **Direct bilirubin** |  |  |  |  |  |  |  |  |
| **All population** | -0.11%(-0.13%,-0.09%) | < 2.00×10^-16^ | 0.997 (0.979,1.015) | 0.721 | 0.12%(0.05%,0.18%) | 0.000341 | 0.939 (0.921,0.957) | 1.46×10^-10^ |
| **Older adults** | 0.04%(-0.02%,0.09%) | 0.177846 | 1.162 (1.107,1.219) | 0.04057 | 0.45%(0.28%,0.62%) | 1.89×10^-7^ | 0.976 (0.929,1.026) | 0.343 |
| **Women adults** | -0.07%(-0.10%,,-0.04%) | 2.20×10^-7^ | 1.172 (1.086,1.266) | 4.95×10^-5^ | -0.02%(-0.05%,0.01%) | 0.225 | 0.876 (0.849,0.903) | < 2.00×10^-16^ |

Supplementary table 6. Association analyses of circulating bilirubin and specific deficit risk.

| **Outcomes** | **Exposure** | **OR** | **95% CI** | ***P*** |
| --- | --- | --- | --- | --- |
| Sensory | Total bilirubin | 0.959 | 0.942-0.977 | <0.001 |
|  | Direct bilirubin | 0.976 | 0.957-0.996 | 0.017 |
| Cranial | Total bilirubin | 0.910 | 0.895-0.926 | <0.001 |
|  | Direct bilirubin | 0.964 | 0.946-0.981 | <0.001 |
| Mental wellbeing | Total bilirubin | 0.857 | 0.830-0.885 | <0.001 |
|  | Direct bilirubin | 0.961 | 0.929-0.994 | 0.022 |
| Infirmity | Total bilirubin | 0.889 | 0.874-0.904 | <0.001 |
|  | Direct bilirubin | 1.062 | 1.043-1.081 | <0.001 |
| Cardiometabolic | Total bilirubin | 1.145 | 1.124-1.165 | <0.001 |
|  | Direct bilirubin | 1.593 | 1.563-1.624 | <0.001 |
| Respiratory | Total bilirubin | 0.852 | 0.836-0.869 | <0.001 |
|  | Direct bilirubin | 0.920 | 0.901-0.939 | <0.001 |
| Musculoskeletal | Total bilirubin | 0.859 | 0.837-0.882 | <0.001 |
|  | Direct bilirubin | 0.951 | 0.925-0.979 | <0.001 |
| Immunological | Total bilirubin | 0.921 | 0.904-0.939 | <0.001 |
|  | Direct bilirubin | 0.895 | 0.877-0.914 | <0.001 |
| Cancer | Total bilirubin | 0.904 | 0.877-0.931 | <0.001 |
|  | Direct bilirubin | 0.922 | 0.893-0.953 | <0.001 |
| Pain | Total bilirubin | 0.888 | 0.873-0.903 | <0.001 |
|  | Direct bilirubin | 0.942 | 0.925-0.959 | <0.001 |
| Gastrointestinal | Total bilirubin | 0.875 | 0.849-0.901 | <0.001 |
|  | Direct bilirubin | 0.943 | 0.913-0.974 | <0.001 |

Supplementary table 7. Linear Mendelian randomization analyses of genetically predicted bilirubin and overall and by stratified residual measured bilirubin categories.

| **Bilirubin** | **Total bilirubin & Frailty index** | | **Direct bilirubin & Frailty index** | |
| --- | --- | --- | --- | --- |
|  | **β (se)** | ***P*** | **β (se)** | ***P*** |
| **Non-stratified** | -0.0000987 (0.000123) | 0.42 | -0.000174 (0.000123) | 0.16 |
| **Residual categories** |  |  |  |  |
| quartile 1 | -0.000937 (0.000258) | 0.0003 | -0.00149 (0.000274) | 5.41×10^-8^ |
| quartile 2 | -0.0000336 (0.000255) | 0.93 | -0.00107 (0.000267) | 6.02×10^-5^ |
| quartile 3 | -0.000208 (0.000251) | 0.41 | -0.0000476 (0.000282) | 0.87 |
| quartile 4 | -0.000999 (0.000236) | 2.19×10^-5^ | -0.000593 (0.000250) | 0.018 |

Notes: β per log-transformed increase in genetically predicted total bilirubin or direct bilirubin.

Both were adjusted for age, sex, assessment centers, SNP array, top 10 genetic principal components.

**Supplementary table 8.** Linear Mendelian randomization estimates from robust methods (MR‐Egger and weighted median)

|  | Total bilirubin & Frailty index | |  | Direct bilirubin & Frailty index | |
| --- | --- | --- | --- | --- | --- |
|  | β (se) | *P* |  | β (se) | *P* |
| WGSs method | -0.0000987 (0.000123) | 0.42 |  | -0.000174 (0.000123) | 0.16 |
| MR-Egger method | -0.000103 (0.000114) | 0.37 |  | -0.000123 (0.000109) | 0.12 |
| Weighted median method | -0.000112 (0.000185) | 0.66 |  | -0.000155 (0.000177) | 0.56 |
